# *Human papillomavirus* and Its Association with Other Sexually Transmitted Coinfection among Sexually Active Women from the Northeast of Brazil

**DOI:** 10.1155/2020/8838317

**Published:** 2020-10-30

**Authors:** Ana Paula Almeida Cunha, Ilka Kassandra Pereira Belfort, Francisco Pedro Belfort Mendes, Gerusinete Rodrigues Bastos dos Santos, Lucas Henrique de Lima Costa, Pablo de Matos Monteiro, Renata Lemos Gaspar, Mariele Borges Ferreira, Alice de Sá Ferreira, Sally Cristina Moutinho Monteiro, Flávia Castello Branco Vidal

**Affiliations:** ^1^Federal University of Maranhão, São Luís, Brazil; ^2^Department of Pharmacy, Federal University of Maranhão, São Luís, Brazil; ^3^Department of Morphology, Federal University of Maranhão, São Luís, Brazil

## Abstract

**Objective:**

To verify the association between HPV infection and the presence of coinfections (*Chlamydia trachomatis*, *Trichomonas vaginalis*, and *Neisseria gonorrhoeae*) in women in the state of Maranhão.

**Methods:**

HPV-DNA detection was performed by the nested PCR, using the primers PGMY09/11 and GP + 5/GP + 6. For the identification of sexually transmitted agents, conventional PCR was performed using the following primers: KL1/KL2 (*Chlamydia trachomatis*), TVA5/TVA6 (*Trichomonas vaginalis*), and HO1/HO3 (*Neisseria gonorrhoeae*). DNA-HPV positive samples were subjected to automated sequencing for genotyping.

**Results:**

Among the 353 women evaluated, 204 (57.8%) had HPV-DNA, of which 140 (68.6%) exhibited HPV/STIs, while 64 (31.4%) had the only HPV. *T. vaginalis* infection showed a positive association with HPV (*p*=0.003). Women without cervical lesions were predominant (327/92.6%); however, the largest number of lesions was reported in women who had HPV/coinfections (18/8.8%). Multiple regression analysis showed that both HPV only and the concomitant presence of HPV/STI were able to indicate the occurrence of epithelial lesions (*R* = 0.164; *R*2 = 0.027).

**Conclusion:**

The findings suggest that the presence of *T. vaginalis* can contribute to HPV infection, and HPV/IST association may influence the development of cervical intraepithelial lesions that are precursors of cervical cancer.

## 1. Introduction

*Human papillomavirus* (HPV) is one of the most prevalent sexually transmitted infections (STIs) worldwide, both in men and women, infecting about 80% of the world's sexually active population [1]. More than 150 viral types have been identified, and at least 13 types have the potential to cause persistent infection and the progression of cervical lesions [2].

Although most cases of HPV infection are asymptomatic and transient, some viral types, such as HPV 16 and 18, are associated with an increased risk of developing anogenital cancer in men and women, including cervical cancer and penile cancer [1]. Among women, cervical cancer is the fourth most common type, with 99% of cases associated with HPV infection [3].

However, high-risk HPV infection is not a sufficient cause for the development of the carcinogenic process [4]. Other sexually transmitted infections (STIs) also represent a public health problem, with more than 1 million people infected daily in the world, and can contribute to the progression of cervical lesions [5]. They usually cause a diversity of symptoms in women and are associated with high rates of morbidity and mortality in sexually active individuals [5].

The relationship between HPV infection and other microorganisms plays an important role in the progression of cervical lesions to cancer [6]. Interactions between HPV and other microorganisms, such as *Chlamydia trachomatis, Trichomonas vaginalis*, and *Neisseria gonorrhoeae*, may contribute to the damages caused by HPV, as well as to persistent infection and cancer progression [7–9].

The main factor associated with the presence of coinfections is the chronic inflammation caused by these microorganisms, which can act as a promoter of carcinogenesis by inducing the occurrence of significant cellular and molecular changes. The proliferation, recruitment of inflammatory cells, and increased production of reactive oxygen species (ROS) during the inflammatory process can lead to damage and inhibition of DNA repair [7]. These changes induced by STIs make the cervical epithelium more susceptible to mutations by activating oncogenes, inactivating tumor suppressor proteins and, thus, facilitating the action of HPV in the induction of tumor lesions [7, 10].

Accurate estimates of the prevalence of HPV and coinfections are limited, especially in the general population. However, epidemiological data are essential for the development of effective strategies for the prevention and early diagnosis of HPV and other STI, as well as for the prevention of cervical cancer. Thus, we sought to evaluate the association between HPV infection and the presence of coinfections (*Chlamydia trachomatis, Trichomonas vaginalis*, and *Neisseria gonorrhoeae*) in women attending the public health system (SUS) in the state of Maranhão, Brazil.

## 2. Methods

### 2.1. Study Design

This is a cross-sectional and noninterventional study of 353 sexually active women who used the public health system of gynecological care in São Luís, Maranhão, Brazil, from March/2018 to March/2019. All individuals answered a semistructured questionnaire based on validated instruments that assessed sociodemographic characteristics, sexual behaviors, smoking status, and history of STIs.

The following exclusion criteria were applied: pregnant women or at less than 45 days postpartum, who had undergone a hysterectomy or were menstruated on the day of the consult. The study was approved by the Federal University of Maranhão Ethics Committee (number 2.383.604). All participants provided written informed consent.

### 2.2. Cervical Cytology

Conventional cytological smears were obtained with Ayre's spatula (ectocervical sample) and endocervical brush (endocervical sample), extended in a glass slide, fixed with ethanol, and stained using the Pap technique. Cytological examinations of Pap smear were reported using the 2001 Bethesda Reporting System.

### 2.3. Sample Collection and DNA Extraction

Samples were collected and placed in the HC2 DNA collection buffer (QIAGEN, CA, USA), and frozen at −20°C until processed. DNA/HPV extraction was performed by using QIAamp DNA Mini and Blood Kit (QIAGEN, CA, USA) according to manufacturer instructions. Total DNA was isolated, eluted in 100 mL AE buffer, and stored at −20°C. Extracted DNA was quantified using the NanoVue system (GE Healthcare Life Sciences, Little Chalfont, UK).

### 2.4. HPV Detection

The presence of DNA/HPV was detected using nested polymerase chain reaction (nested PCR) with the primer sets PGMY09/11 (first-round PCR) and GP + 5/GP + 6 (second-round PCR) using the Platinum™ Taq DNA Polymerase system (Invitrogen™, NY, USA) [11]. All samples were submitted for *β*-globin amplification.

The first-round mix was formed by 25 *μ*L of the final volume, containing 10 pmol of each primer, 10X reaction buffer containing 1 mM MgCl2, 10 mM dNTP, 1 U of Taq Polymerase Platinum (Invitrogen), and 100 ng of genomic DNA [11]. The amplification cycles consisted of initial denaturation for 2 minutes at 95°C followed by 40 denaturation cycles for 40 seconds at 95°C, 40 seconds of annealing at 55°C, and 40 seconds of extension at 72°C.

The second-round (GP + 5/GP + 6) consisted of an initial denaturation at 95°C for 4 minutes followed by 45 cycles of denaturation at 95°C for 45 seconds, annealing at 40°C for 1 minute, and extension at 72°C for 1 minute. The reaction mix was formed by 25 *μ*L of the final volume, containing 10 pmol of each primer, 10X reaction buffer, 1 mM of MgCl2, 10 Mm of dNTP, 2U of Taq Polymerase Platinum (Invitrogen), Milli-Q water, and 100 ng of genomic DNA [11].

### 2.5. STI Detection

The presence of *Trichomonas vaginalis, Neisseria gonorrhoeae*, and *Chlamydia trachomatis* was detected using polymerase chain reaction (PCR) using the primers suggested by (2013): TVA5/6, HO1/HO3, and KL1/KL2, respectively and the SuperMix Brazil system (Thermo Fisher, Brazil).

The reaction consisted of a final volume of 50 *μ*L, with 41 *μ*L of SuperMix, 10°pmol of each primer, and 100 ng of genomic DNA. The amplification cycles varied according to the primer used. For amplification of *T. vaginalis*, the reaction occurred with an initial denaturation at 94°C for 5 minutes, 45 cycles of 94°C for 30 seconds, annealing at 47°C for 1 minute and extension at 72°C for 45 seconds, and a final extension cycle at 72°C for 10 minutes. For amplification of *C. trachomatis*, the reaction occurred with an initial denaturation at 94°C for 5 minutes, 35 cycles of 94°C for 30 seconds, annealing at 55°C for 1 minute and extension at 72°C for 45 seconds, and a final extension cycle at 72°C for 10 minutes. For amplification of *N. gonorrhoeae*, the reaction occurred with an initial denaturation at 94°C for 5 minutes, 40 cycles of 94°C for 30 seconds, annealing at 55°C for 1 minute and extension at 72°C for 45 seconds, and a final extension cycle at 72°C for 10 minutes.

### 2.6. HPV Genotyping

PCR products amplified were purified using the Genomic DNA Purification Kit (Sigma-Aldrich, Missouri, USA), labeled using the Big Dye Terminator v3.1 Cycle Sequencing Kit (Applied Biosystems, Foster City, CA), and analyzed using an ABI Prism 3130XL Genetic Analyzer (Applied Biosystems). The sequences were edited and analyzed using 4Peaks Software (Nucleobytes, Amsterdam, Netherlands). HPV genotypes were identified using the BLASTn (Basic Local Alignment Search Tool, http://blast.ncbi.nlm.nih.gov/).

### 2.7. Data Analysis

The data considered categorical were presented in proportion, using the X^2^ (Chi-square) or Fisher's exact tests to assess the association between HPV/STI infection and sociodemographic and clinical factors. To assess the risk factors associated with HPV and their significance, the odds ratio test was used. To verify correlations and predictive variables for the appearance of cervical lesions, binary logistic regression and odds ratio were performed. A 95% confidence interval was adopted for all analyzes performed, and the data were considered statistically significant when *p* values ≤ 0.05. All tests were performed using IBM SPSS® Statistics version 24.0.

## 3. Results

**Figure 1:**
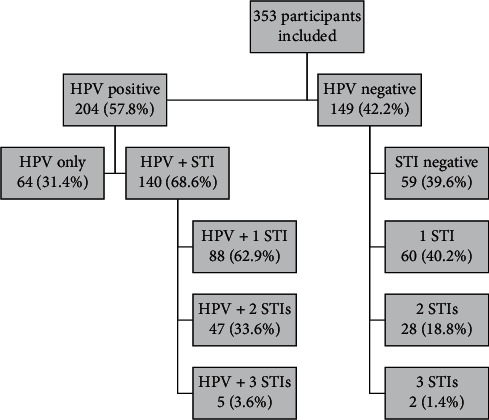
Fluxogram of 353 sexually active women included in the study. HPV: *Human papillomavirus*; STI: sexually transmitted infection.

In total, 353 women (age average 39.75, SD ± 13.85) who attended public primary healthcare were included in the study. The population profile (Table 1) was women with schooling up to complete/incomplete high school (54.7%), self-declared brown color (61.5%), and having a fixed partner (51.2%). Regarding sexual habits and risk factors, most women reported menarche before 13 years of age (57.8%), the first sexual intercourse after 15 years of age (62.9%), not using condoms during sexual intercourse (70.8%), had up to 3 sexual partners during life (62.3%), had up to 3 pregnancies (70.3%), and declared as a nonsmoker (73.9%). Among the participants, 204 (57.8%) were positive for DNA-HPV, of which 140 (68.6%) exhibited HPV and other sexually transmitted infections (STIs), and 64 (31.4%) had only HPV. Among women who exhibited HPV/STI coinfection, 1 STI in addition to HPV was found in 88 (62.9%) of them (Figure 1). In women who did not show HPV, 59 (39.6%) presented infection with at least one of the evaluated STIs. And in those who did not show HPV infection, 59 (39.6%) had no other STIs, and 60 (40.2%) had at least 1 STI.

**Figure 2:**
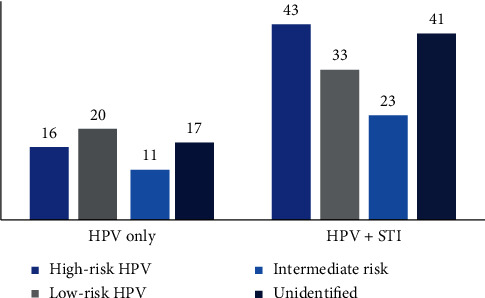
Distribution of HPV viral types according to the oncogenic potential in women with single HPV infection and women with HPV/STIs (*C. trachomatis*, *N*. *gonorrhoeae*, and *T. vaginalis*).

Figure 2 shows the distribution of cases of HPV infection and STIs according to the viral type identified. There was a high prevalence of high oncogenic risk HPV among women who had coinfections; however, this result is not statistically significant. Among women who exhibited single HPV infection, 20 (31.3%) had viral types of low oncogenic risk.

Sociodemographic data and socioeconomic aspects, such as age, skin color, income, and educational level, were similar among women who had only HPV, women with HPV/STI coinfections, and women who were HPV negative (Table 1). Despite most women reported low socioeconomic indexes, the monthly income showed statistically significant values (*p* < 0.001), indicating that women with less than 1 minimum wage had higher risk of presenting HPV/coinfections. No statistically significant differences were found between the characteristics related to sexual life habits in the groups, such as condom use during sexual intercourse, the number of partners, age at menarche, and first sexual intercourse.

The odds ratio analysis showed that *T. vaginalis* infection was positively associated with HPV (*p*=0.003) (Table 2). Thus, participants with *T. vaginalis* infection were 2.081 times more likely to present HPV compared to women who did not have the infection. Similarly, menopause was statistically associated with HPV infection, in addition to a cause and effect relationship. That is, women who are in menopause are 1.72 more likely to develop HPV infection when compared to women who still have a menstrual cycle.

The number of sexual partners and pregnancy were protective factors regarding the presence of HPV infection. These data mean that women who had fewer sexual partners and pregnancies had 0.033 and 0.301 fewer risks of presenting HPV, respectively, compared to women who reported having a higher number of partners and a higher number of pregnancies.

Regarding cervical alterations, 327 (92.6%) women had no cervical lesions identified in the Pap test, both among those with HPV only and among those who had HPV and other associated STIs. Despite the reduced number of cervical lesions among the study participants, only 26 (7.4%), the largest number of alterations was reported in women who had HPV and coinfections (18/8.8%). The identified changes are atypical squamous cells of undetermined significance (ASC-US, 10/2.84%), low-grade squamous intraepithelial lesion (LSIL, 8/2.26%), and atypical squamous cells that cannot exclude high-grade intraepithelial lesion (ASC-H, 6/1.70%).

Binary logistic regression analysis verified that the isolated presence of HPV, *N. gonorrhea*, *T. vaginalis*, *C. trachomatis*, and HPV/STI concomitantly is contributing factors for the development of cervical lesions (Table 3). The model containing these predictors was significant (X^2^ (5 degrees of freedom) = 61.63; *p* < 0.001, *R*2 = 0.260); however, only the concomitant presence of HPV/STI was a factor that significantly contributed to cervical lesion development (OR: 12.63; 95% CI: 4.750–33.632). The associated presence of HPV and STIs had a positive correlation with the presence of cellular changes, which means that as the number of women infected by HPV/STIs increases, the number of cervical lesions also increases. In addition, women with HPV/STIs coinfection represent 12.63 times more likely to develop cervical alterations.

## 4. Discussion

In this study, data from sexually active women were used to assess the presence of HPV and other sexually transmitted infections (STIs), as well as the association between them for the progression of cervical lesions. The study participants were women seeking for cervical cancer screening in basic health units located in regions of difficult access and social vulnerability.

HPV infection was highly prevalent in the study population, and among these, HPV associated with another STI was more prevalent than HPV infection alone. *T. vaginalis* showed a positive association with the presence of HPV, showing that this parasite increased the risk of women presenting HPV infection. Mercer and Johnson (2018) suggested that the nitrosamines produced by *T. vaginalis* promote cervical inflammation, thus causing wounds that allow HPV to penetrate the basal layer of the cervical epithelium. Other studies also indicate the association between *T. vaginalis* and the increased risk of cervical intraepithelial lesions [12–14]; however, this fact was not observed in the present study.

Regarding HPV classification, high oncogenic risk types were more prevalent in women who had coinfections (HPV/STIs), while low oncogenic risk types were generally found alone. These data suggest that, due to its different characteristics of aggressiveness, the viral type may have contributed to the presence of other STIs in the cervical epithelium, although these data were not statistically significant. In this context, women with concomitant infection by high-risk HPV and other STIs, such as *T. vaginalis*, *C. trachomatis*, and *N. gonorrhoeae*, may have promoted more aggressive changes in the cervical microenvironment, thus making it difficult for the immune system to eliminate these pathogens.

Studies aiming to assess the prevalence of STIs in the general population are mainly focused on the identification of HIV and syphilis [15–17]. Despite the number of studies aiming to evaluate the prevalence of multiple STIs, only a few sought to assess the association between HPV and other STIs with the progression of cervical lesions [2, 17]. Since there are currently more than 30 types of STIs worldwide and different methods of detecting these pathogens, we need to be careful in comparing studies. In addition, the type of population may also vary among studies, with most of them being performed in pregnant women, HIV-positive women, sex workers, homosexuals, or injecting drug users [18].

A study performed in South Africa evaluated the presence of HPV and five other STIs, namely, herpes simplex type 2 (HSV-2), *C. trachomatis*, *N. gonorrhoeae*, and *T. pallidum* in women between 16 and 24 years of age. They found that 47% of women had HPV and two STIs, 36% exhibited HPV and 1 STI, and 17% had only HPV; however, no statistically significant association was found between the infections [9].

Similarly, Magaña-Contreras et al. [19] assessed the prevalence of HPV and other 4 STIs (*C. trachomatis*, *Gardnerella vaginalis*, *Mycoplasma genitalium*, and *Ureaplasma* spp.) in women seeking routine gynecological care in Italy. They identified that HPV was associated with 1 STI (33.8%), and the highest prevalence rate was attributed to *Ureaplasma* spp. [19].

A multicenter study performed in Brazil by Kops et al. [2] stated that HPV-isolated infection was higher than HPV/coinfections. Among women who reported HPV/coinfections, women with one secondary infection were predominant, primarily by *N. gonorrhoeae* and HIV. Despite being a study carried out in a population of Brazilian women, the results differ from those reported in our study [2]. A possible explanation may be the method used for STI detection used by Kops et al. [2], which occurred through the participants' self-report and may not reflect the actual status of these STIs during the interview.

Despite presenting similar values regarding HPV prevalence and HPV/STI association, these studies present important differences. The main differences are the sample number, the types of STI evaluated, and the molecular techniques used, which may have different specificity in the detection of pathogens. Thus, to fully understand the influence of multiple STI for the persistence of HPV and the progression of cervical lesions, it is necessary to optimize techniques that allow the simultaneous detection of a greater number of STIs.

Multiple STIs have the potential to influence HPV infection, although literature data report that *C. trachomatis* and *T. vaginalis* are among the main infections associated with HPV virus. Similarly, studies report that *C. trachomatis* contributes to the acquisition of high-risk HPV viruses; however, the present study did not observe an association between these two pathogens [7, 20–22]. We suggested that there is a possible cooperation between multiple microorganisms, contributing to cellular microenvironment changes that facilitate HPV infection. Further analysis is needed to assess the potential of these pathogens individually and their role in HPV infection and lesion progression.

Menopause also demonstrated a positive association with the presence of HPV, as well as a cause/effect relationship. This means that women in menopause were 1.72 times more likely to have a viral infection, compared to women who still have menstrual cycles. Hormonal changes caused by menopause can lead to the reactivation of latent infections or, in cases of women with active sex life, contribute to the acquisition of a new infection [23]. A high prevalence of women who reported having a steady partner also reported not using condoms, both in women with HPV infection only and in women with HPV/coinfections. Although these data were not statistically significant, it may have contributed to the high number of HPV and STIs in this population. Women in menopause and who have a steady partner tend to have a perception that they are protected against STIs and, therefore, do not use condoms in all sexual intercourse. In addition, the occurrence of unprotected sexual intercourse in extramarital relationships can favor the transmission of STIs to partners.

The number of sexual partners and parity represented protective factors against HPV infection, which means that women with fewer partners and fewer pregnancies had a reduced risk of contracting HPV. The high number of sexual partners and the early onset of sexual activity are established risk factors for increasing the risk of acquiring multiple sexually transmitted infections during life [24]. Similar to the present study, Mbulawa et al. [24] reported that women who had at least one pregnancy or had from 4 to 10 sexual partners during a lifetime were more likely to have an HPV infection. The high number of sexual partners increases the contact with multiple sexually transmitted pathogens, evidencing that screening for STIs and awareness programs should include both men and women.

Multiple regression analysis verified that HPV infection only and the concomitant infection of HPV and other STIs were associated with cervical epithelial lesions identified in the Pap smear among the studied participants. This means that as the number of women infected with HPV and STIs (*C. trachomatis*, *N. gonorrhoeae*, and *T. vaginalis*) increases, so does the number of women with cervical lesions. These data corroborate with previous studies, showing that the presence of multiple infections favors the persistence of HPV in the cervical epithelium, thus influencing the progression of lesions [12, 23, 25, 26].

However, despite the high rates of infections both by HPV and other STIs in the study population, these factors did not reflect in a high number of cervical lesions. The reduced number of cervical cancer precursor lesions, in contrast to the high prevalence of HPV, *T. vaginalis*, *C. trachomatis*, and *N. gonorrhoeae* among the women evaluated, supports the hypothesis that, despite being configured as an important precursor to cervical oncogenesis, the presence of HPV alone is not sufficient for cancer development. Other factors such as persistence and oncogenic viral type, the expression of viral oncogenes, and the integration of the viral genome in the host cell also have an important influence on cervical carcinogenesis [27].

It is important to highlight that persistent HPV infection is closely associated with HPV-related cytological alterations; however, since this was a cross-sectional study, these data were not assessed. In this sense, although our findings indicate that the presence of HPV-STI coinfection contributes to the development of cervical changes, future studies are crucial to investigate the role of these coinfections to both contributing to viral persistence and the progression of precancer cervical lesions (De Sanjosé; Brotons; Pavón, 2018; Doorbar, 2016; Verteramo et al., 2009). This limitation could be solved with longitudinal studies, which could evaluate the ability of women to eliminate the HPV virus as well as other STIs in a given period of time, and the subsequent status regarding cervical alterations.

The presence of coinfections associated with HPV has been reported in other studies, due to its association with the progression of cervical intraepithelial lesions. However, the microorganisms detected vary in each study. In women with HPV, the presence of coinfections by *C. trachomatis*, *Ureaplasma urealyticum*, *Trichomonas vaginalis*, and *Neisseria gonorrhoeae* are among the main STIs identified [7, 8, 17, 22, 24, 28].

A study performed in Brazil [1] sought to evaluate the presence of HPV and coinfections in 132 women with or without cervical lesions. They identified that stable union, HPV infection, *T. vaginalis*, and *Gardnerella vaginalis* were associated with the occurrence of cervical lesions; however, the study did not evaluate the influence of the concomitant presence of HPV and other infections for the development of cervical lesions. A similar study performed by Lima et al. [8] demonstrated an association between HPV and CIN (cervical intraepithelial neoplasia), as well as *C. trachomatis* and CIN, but the concomitant infection by these two pathogens was not associated with cervical lesions.

The presence of coinfections can reduce the immune system's ability to eliminate HPV infection, in addition to favoring the progression of cervical lesions through the unregulated expression of viral oncogenes [29]. STIs can also induce a chronic inflammatory process, promoting the secretion of immune mediators that increase the production of reactive oxygen species that can, consequently, lead to tissue damage and favor both the entry and performance of HPV viral oncogenes [7].

These pathogens can also modify the composition of the vaginal microbiota, contributing to the malfunction of the cervical microenvironment [30]. Combined with the presence of HPV and other sexually transmitted pathogens, factors such as genetic predispositions, sexual behavior, and hygiene habits that differ in different countries and regions can influence the composition of the vaginal microbiota [30]. Low economic status and the adoption of risky sexual practices may have contributed to the high number of HPV infections and other STIs in the study population.

## 5. Conclusion

This study provides information on HPV and other STI association in women in the Northeast of Brazil through the molecular detection of these pathogens. The Northeast region, especially the State of Maranhão, has low socioeconomic rates that contribute to the high rates of cervical cancer and sexually transmitted infections in this population.

The findings suggest that *T. vaginalis* may favor HPV infection, and the HPV/STI association may contribute to the development of cervical intraepithelial lesions that are precursors of cervical cancer. Importantly, our study could not evaluate the presence of persistent HPV infection among the participants, which could have contributed to a better understanding about the role of these STIs in the natural history of HPV infection as well as in the development of precursor cancer lesions. These STIs, including HPV, are an important public health problem since they are related to the development of multiple urogenital complications, such as infertility and cancer. Although the association between HPV and cervical cancer is well established, further studies regarding HPV/STIs need to be performed in order to understand the multiple and complex factors that may be involved in cervical carcinogenesis.

## Figures and Tables

**Table 1:** Sociodemographic characteristics of the participants according to the presence of HPV and STIs.

Variable		Total		HPV positive				HPV negative				*p* value
				STI positive (*N* = 140)		STI negative (*N* = 64)		STI positive (*N* = 90)		STI negative (*N* = 59)		
		*n*	%	*n*	%	*n*	%	*n*	%	*n*	%	
Age	18–24 years	63	17.8	27	19.3	11	17.2	16	17.8	9	15.2	0.400
	25–34 years	76	21.5	28	20	10	15.6	22	24.4	16	27.1	
	35–44 years	87	24.6	29	20.7	17	26.5	28	31.1	13	22.03	
	45–54 years	67	19	28	20	12	18.7	17	18.8	10	17	
	≥55 years	60	17.1	28	20	14	21.8	7	7.8	11	18.7	

Monthly income	<1 minimum wage	204	57.8	95	67.8	37	57.8	51	56.6	21	35.6	0.001
	>1 minimum wage	149	42.2	45	32.1	27	42.1	39	43.3	38	64.4	

Education level	Elementary school complete/incomplete	117	33.1	54	38.6	18	28.1	30	33.3	15	25.4	0.600
	Secondary school complete/incomplete	193	54.7	66	47.1	38	59.3	52	57.8	37	62.8	
	Graduate school complete/incomplete	34	9.7	16	11.4	6	9.3	7	7.8	5	8.4	
	Illiterate	9	2.5	4	12.1	2	3.1	1	1.1	2	3.4	

Skin color	White	27	7.6	10	7.1	5	7.8	9	14.06	3	5.08	0.200
	Brown	217	61.5	89	63.6	38	59.3	50	55.5	40	67.8	
	Black	100	28.3	40	28.6	20	31.2	25	27.8	15	25.4	
	Yellow	9	2.57	1	0.7	1	1.5	6	2.6	1	1.7	

Marital status	With partner	181	51.27	78	55.7	28	43.7	49	54.4	26	44.1	0.200
	Single	172	48.7	62	44.3	36	56.2	41	45.6	33	55.9	

Menarche	Before 13 years old	204	57.8	76	54.3	38	59.3	52	57.8	38	64.4	0.600
	Before 13 years old	149	42.2	64	45.7	26	40.6	38	42.2	21	35.6	

First sexual intercourse	Before 15 years old	131	37.1	52	37.1	20	31.2	34	37.8	25	42.3	0.600
	After 15 years old	222	62.9	88	62.9	44	68.7	56	62.2	34	57.6	

Condom use	Yes	103	29.2	43	30.7	18	28.1	28	31.1	14	23.7	0.700
	No	250	70.8	97	69.3	46	71.8	62	68.9	45	76.3	

Number of partners	Up to 3	220	62.3	95	67.8	39	60.9	56	62.2	30	50.8	0.100
	More than 3	133	37.7	45	32.1	25	39.06	34	37.8	29	49.2	

Number of pregnancy	Up to 3	248	70.3	98	70	43	67.2	69	76.6	38	64.4	0.300
	More than 3	105	29.7	42	30	21	32.8	21	23.3	21	35.6	

Tobacco use	Yes	27	7.7	14	10	3	4.7	9	10	1	1.7	0.200
	No	261	73.9	96	68.6	50	78.1	66	73.3	49	83	
	Ex-smoker	65	18.4	30	21.4	11	17.2	15	10.7	9	15.3	

**Table 2:** Risk factors for HPV presence in women according to odds ratio and confidence interval (95% CI).

	Odds ratio	CI (95%)	*p* value
*N. gonorrhoeae*	1.339	0.828–2.164	0.20
*T. vaginalis*	2.081	1.283–3.377	0.03
*C. trachomatis*	0.791	0.505–1.239	0.30
Smoking	1.376	0.854–2.242	0.10
Alcohol consumption	1.038	0.680–1.586	0.80
Number of partners	0.033	0.014–0.078	<0.001
Menopause	1.72	1.070–2.794	0.02
Pregnancy	0.301	0.175–0.518	<0.001

*X* ^2^ (Chi-square) or Fisher's exact test (CI: 95%). Odds ratio was considered statistically significant when *p* < 0.05.

**Table 3:** Binary logistic regression to verify possible predictors of cytological lesions.

	Odds ratio	CI (95%)	Wald test	*p* value
*HPV*	0.495	0.184–1.334	1.934	0.1
*N. gonorrhoeae*	1.298	0.664–2.536	0.583	0.4
*T. vaginalis*	1.509	0.783–2.911	1.509	0.2
*C. trachomatis*	0.623	0.324–1.198	1.63	0.1
HPV + STI	12.639	4.750–33.632	25.807	<0.001

HPV: *Human papillomavirus*; STI: sexually transmitted infection; data were analyzed through multiple linear regression, using one-way ANOVA to verify statistical significance (*p* < 0.05).

## Data Availability

Data are available within the article.

## Additional Points

In brief, this study provides information on HPV and other STI association in women in the Northeast of Brazil through the molecular detection of these pathogens. Our findings suggest that *T. vaginalis* may favor HPV infection and that HPV/STI association may influence the development of cervical cancer precursor lesions.
